# The mechanistic pathways of oxidative stress in aortic stenosis and clinical implications

**DOI:** 10.7150/thno.71813

**Published:** 2022-07-04

**Authors:** Kailun Phua, Nicholas WS Chew, William KF Kong, Ru-San Tan, Lei Ye, Kian-Keong Poh

**Affiliations:** 1 Department of Cardiology, National University Heart Centre, National University Hospital, Singapore, Singapore; 2 Department of Cardiology, National Heart Centre Singapore, Singapore, 169609, Singapore; 3 National Heart Research Institute Singapore, National Heart Centre Singapore, Singapore, 169609, Singapore; 4 Yong Loo Lin School of Medicine, National University of Singapore, Singapore

**Keywords:** Severe aortic stenosis, reactive oxidative species, oxidative stress

## Abstract

Despite the elucidation of the pathways behind the development of aortic stenosis (AS), there remains no effective medical treatment to slow or reverse its progress. Instead, the gold standard of care in severe or symptomatic AS is replacement of the aortic valve. Oxidative stress is implicated, both directly as well as indirectly, in lipid infiltration, inflammation and fibro-calcification, all of which are key processes underlying the pathophysiology of degenerative AS. This culminates in the breakdown of the extracellular matrix, differentiation of the valvular interstitial cells into an osteogenic phenotype, and finally, calcium deposition as well as thickening of the aortic valve. Oxidative stress is thus a promising and potential therapeutic target for the treatment of AS. Several studies focusing on the mitigation of oxidative stress in the context of AS have shown some success in animal and *in vitro* models, however similar benefits have yet to be seen in clinical trials. Statin therapy, once thought to be the key to the treatment of AS, has yielded disappointing results, however newer lipid lowering therapies may hold some promise. Other potential therapies, such as manipulation of microRNAs, blockade of the renin-angiotensin-aldosterone system and the use of dipeptidylpeptidase-4 inhibitors will also be reviewed.

## Introduction

Aortic stenosis (AS) is one of the most prevalent valvular heart diseases and constitutes a large portion of the burden on public health globally 1. While degenerative disease is the most common aetiology of AS, other causes include congenital heart diseases, rheumatic heart disease. Moreover, systemic inflammatory and infiltrative disease can also contribute to AS development and progression 2. Despite our understanding of this condition, there is currently no effective medical therapy to reduce the symptoms, mortality or valve progression of degenerative AS. The mainstay treatment of symptomatic AS is aortic valve replacement, either surgical (SAVR), or more recently, transcatheter aortic valve implantation (TAVI) in select patient populations 3. SAVR and TAVI lead to better outcomes in survival and hospitalisations compared to medically managed patients with severe AS, with improved symptomology and ventricular function 3.

The aortic valve normally consists of three leaflets, each having a trilaminar matrix composing of ordered layers of collagen fibres, proteoglycans and elastic fibres respectively, bestowing compliance during systole, and the ability for leaflet apposition during diastole to prevent the backflow of blood 4. The aortic valve is composed of three layers - the ventricularis, spongiosa and fibrosa. The ventricularis on the ventricular side of the leaflet contains fibres rich in elastin, while the fibrosa on the aortic side of the leaflet comprises collagen fibres. These fibres give the aortic valve flexibility and strength to withstand decades of repetitive movement. The spongiosa consists of an extracellular matrix rich in glycosaminoglycans, and valvular interstitial cells (VICs, also called cardiac fibroblasts), are the predominant cell type in all layers 5.

The pathophysiology of degenerative AS is a complex interplay of inflammation, lipid infiltration and fibro-calcification 6. Histological studies on calcified aortic valve tissues reveal inflammatory infiltrates characterised by macrophages and T-cells, while elevated levels of pro-inflammatory cytokines have been found in stenotic valves as well 7. Coagulation also plays a part - the expression of coagulation factors such as factor VII and factor X have been found in stenotic aortic valves 8. Thrombin and tissue factor is suspected to be involved in the calcification process of degenerative AS 9. Under pro-inflammatory conditions, VICs will express these factors 5, which may trigger the coagulation cascade and leading to a build-up of fibrin within the valve. In fact, there is a positive correlation between the amount of fibrin in the aortic valve and the transvalvular pressure gradient 10.

These processes lead to progressive thickening of the valve leaflets, compromising leaflet motility and resulting in a narrowed valve orifice. In severe cases, this causes significant systolic blood flow obstruction, increased systemic afterload and ultimately, left ventricular remodelling, cardiac dysfunction, and heart failure 7.

The aetiology of degenerative AS shares many similarities to atherosclerosis. Risk factors such as age, smoking, obesity, hypertension, hypercholesterolaemia and diabetes are linked to the development of degenerative AS 8-15. At the molecular level, stenotic aortic valves resemble aortic plaques and contain elevated levels of matrix metalloproteinases, oxidised phospholipids (oxPLs), and calcium 16-18.

Reactive oxidative species (ROS) are reactive molecules that are produced in the process of oxygen metabolism, and major sources include the mitochondrial electron transport chain as well as the oxidoreductase group of enzymes 15,19. Oxidative stress, an imbalance between ROS such as superoxide and peroxynitrite, and antioxidant protective mechanisms, is one of the major upstream contributors to inflammation, lipid infiltration and calcification, with downstream effects leading to the development of degenerative AS. While oxidative stress affects both the extracellular matrix and the cells of the aortic valve, most of its influence revolves around the VICs (Central Illustration).

Oxidative stress is prevalent in older age, possibly due to increased ROS, lower levels of antioxidants, reduced repair/removal mechanisms as well as the presence of multiple comorbidities such as chronic kidney disease 20. TAVI tends to be offered to this population on patients, compared to younger patients with fewer comorbidities in the SAVR cohort. The alleviation of AS with TAVI causes near-instantaneous decrease in levels of oxidative stress. Higher pre-TAVI baseline level of plasma superoxide has been associated with increased post-interventional inflammation and poorer clinical outcomes 21; these ROS can act as signalling molecules in upregulating pro-osteogenic pathways that will be described later.

Hence, therapies targeting oxidative stress may potentially hold the key to slowing or reversing the progression of degenerative AS. A literature review was conducted on PubMed using the search terms 'aortic stenosis' and 'oxidative stress'. This review will explore the mechanisms of oxidative stress in the development of degenerative AS, as well as discuss the clinical implications and possible medical therapies.

## Oxidative Stress and Lipid Infiltration

The initiation of AS is hypothesized to be related to oscillatory shear stress, causing endothelial dysfunction 15. In particular, bicuspid aortic valves are less efficient in dissipating this shear stress, resulting in accelerated endothelial damage in these phenotypes 22,23. As such, patients with bicuspid aortic valves develop AS at an earlier age and display more rapid progression compared to those with tricuspid aortic valves 14. Once the valve endothelium is damaged, there is infiltration of OxPL-containing lipoproteins such as lipoprotein(a) and oxidised low-density lipoprotein (oxLDL), similar to the pathogenesis of atherosclerosis 24,25. These circulating lipids enter by initially adhering tightly to lysine-binding sites on the exposed valve surfaces 26. Indeed, there is an association between elevated levels of oxPLs with the progression of AS and subsequent requirement for intervention 27. OxPLs are also proapoptotic, and these apoptotic pathways may also be implicated in the pathophysiology of AS 15.

**Figure 1:**
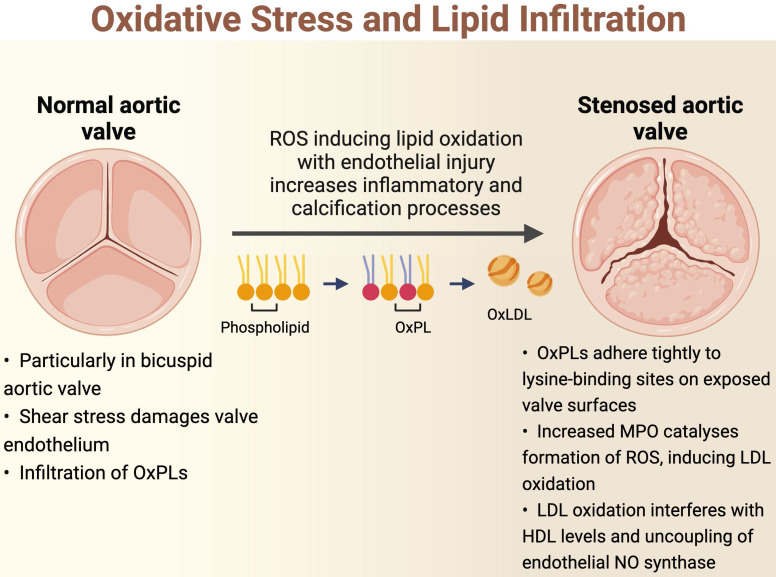
Oxidative Stress and Lipid Infiltration

Oxidation of lipoproteins can be enzymatic or non-enzymatic; the latter is induced by ROS in a process called lipid peroxidation 19. For example, myeloperoxidase (MPO) is an oxidoreductase enzyme that catalyses the formation of ROS and thus has been implicated in the oxidation of LDL 28. High levels of oxLDLs interfere with the protective, antioxidant effects of high-density lipoprotein (HDL) cholesterol; levels of MPO in valve tissue are found to increase along with the severity of AS, while the converse is true for HDL cholesterol 29. Increasing concentrations of oxLDLs also lead to the uncoupling of endothelial nitric oxide synthase, resulting in a switch in the production from nitric oxide, which is a protective antioxidant, to the ROS superoxide 30-33. Further lipid peroxidation and endothelial injury contribute to the inflammatory and calcification processes within the valve (Figure 1). Further studies demonstrating the association between lipid peroxidation and aortic stenosis are summarised in Table 1 34-41.

## Oxidised Phospholipids are linked to Valvular Inflammation and Calcification

The presence of oxPLs and continuing process of lipid peroxidation within valvular tissue leads to a cascade of inflammatory cell infiltrates characterised by macrophages and T-lymphocytes, and the production of pro-inflammatory cytokines such as tumour necrosis factor-alpha (TNF-α), transforming growth factor-beta 1 (TGF-β1) and interleukin-6 (IL-6) 26.

Oxidative stress also directly upregulates transcription factors leading to downstream expression of genes involved in the inflammatory process; subsequently there is positive feedback with the inflammation causing additional ROS generation that eventually promotes endothelial dysfunction and cell death, leading to a vicious circle 29.

The pro-inflammatory cytokines, coupled with a reduction in the availability of nitric oxide, disrupt the balance between the production and breakdown of the extracellular matrix in the valve 31,42. This imbalance lays the foundations for dystrophic microcalcification 43, and the inflammatory stimuli induce VICs to express osteogenic genes such as those found in the bone morphogenetic protein 2 (BMP2) pathway 2. This osteogenic process is not unlike the one observed in normal bone mineralisation. In fact, osteoblast-like cells have been found in calcified aortic valves 31. In an *in vitro* study, treatment with L-Arginine, a precursor to nitric oxide, was found to be effective in preventing the osteogenic differentiation and reducing matrix calcification of VICs obtained from bovine valves 44.

**Figure 2:**
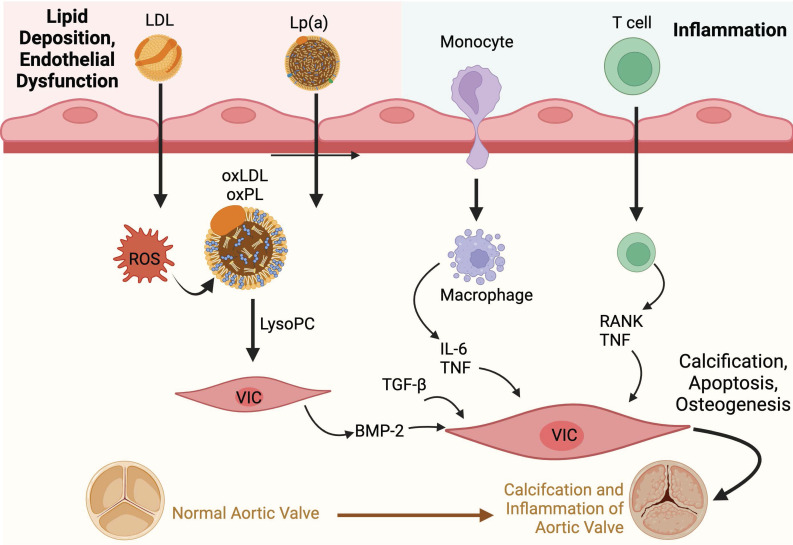
Lipid Deposition and Inflammation

OxPLs activate the toll-like receptors and NF-kB (nuclear factor kappa light chain enhancer of activated B cells) signalling pathway in VICs 2. TNF-α contributes to this process, leading to downstream production of IL-6 (Figure 2). IL-6 then further promotes the expression of the BMP2 and receptor activator of NF-kB ligand superfamily member 11 (RANK)/RANK ligand (RANKL)/osteoprotegerin (OPG) pathways 2. While the RANK/RANKL/OPG pathway promotes osteoclast activity in bones, the opposite is seen in valvular tissues, inducing an osteoblastic phenotype in VICs 14.

Furthermore, OxPLs upregulates alkaline phosphatase, which induces the differentiation of calcifying vascular cells; a similar process may be seen in valvular tissue 27. Similarly, the incubation of aortic VICs with lipoprotein(a) was found to induce osteogenesis, resulting in increased apoptosis as well as a deposition in calcium 45.

The NF-kB pathway also promotes dipeptidylpeptidase-4 (DPP-4) expression, which also induces osteogenesis in VICs by reducing insulin-like growth factor-1 (IGF-1) signalling 46. High DPP-4 expression was found in calcified regions of aortic valves; conversely, levels were negligible in non-calcified regions. Sitagliptin, a DPP-4 inhibitor, was found to inhibit osteogenic changes in VICs *in vitro*, and also reduced aortic valve calcification as well as improved aortic valve indices such as aortic valve area, transaortic peak velocity as well as maximal and mean pressure gradients in animal models 46.

Autotaxin is an enzyme that is both transported in the blood plasma and secreted by several different types of cells, and it is involved in the production of lysophosphatidic acid, which is highly pro-inflammatory 47. Autotaxin is transported into the aortic valve by lipoprotein(a), and is also secreted by VICs 48. It contributes to the inflammatory process and subsequent promotion of osteogenesis via the NF-kB and BMP2 pathways; in fact, it can serve as an independent predictor of calcific AS 49.

## Oxidative Stress Causes Osteogenesis Via Cellular and Mitochondrial Pathways

Besides lipid peroxidation, oxidative stress also directly promotes osteogenesis in valvular tissue via a variety of mechanisms. Endothelial-derived nitric oxide is the endogenous signalling molecule for NOTCH1, a transcriptional regulator that is involved in aortic valve development and inhibits osteogenesis 45,50. The loss of nitric oxide reduces the activity of NOTCH1. There is then downstream activation of Lrp5 (low-density lipoprotein receptor-related protein 5), a protein involved in the osteogenic Wnt/β-catenin pathway 51. Furthermore, ROS directly upregulates the expression of BMP2, which regulates osteogenic differentiation via Runx2 (runt-related transcription factor 2), SMAD1 (Mothers Against Decapentaplegic Homolog 1) and caspase-3 52.

At the cellular level, oxidative stress causes damage to DNA, cellular proteins and lipids, as well as the activation of mitochondrial-driven apoptosis, and the resultant release of apoptotic bodies 15. These apoptotic bodies facilitate the formation of hydroxyapatite crystals, which are nucleation sites for further calcium deposition 14,53. This amorphous epitaxial mineral deposition is independent of osteogenesis and overrides antioxidant effects, causing an exponential increase in the rate of calcification 54.

As mentioned above, mitochondrial electron transport chain complexes I and III are major sources of superoxide degeneration; superoxide ions are converted to the more stable hydrogen peroxide by the superoxide dismutases, and crucially, levels of superoxide dismutases are significantly reduced in calcified regions of valve tissue, resulting in an increase in superoxide 15. *In vivo* treatment of a rabbit model with lipolic acid, which aids the metabolism of hydrogen peroxide, abrogated aortic valve calcification 54. Typically, levels of superoxide dismutases are increased in tissues that are exposed to elevated oxidative stress as an antioxidant defence mechanism, and its expression is found to be elevated in atherosclerotic plaques. Surprisingly however, its activity is reduced in calcified regions of the aortic valve 55.

NADPH (nicotinamide adenine dinucleotide phosphate) oxidase is a key mediator to increased oxidative stress in atherosclerosis, but its significance is debatable in AS. While an earlier landmark study found that its expression and activity was not increased in calcific aortic VICs 16, a more recent *in vitro* study found that specifically, levels of NADPH oxidase 2 are increased in calcific VICs 56. In both *in vitro* and *in vivo* experiments, the same group showed that inhibition of NADPH oxidase 2 significantly reduced calcification in porcine VICs, and improved the rate of change in the aortic valve area, transaortic peak velocity as well as the maximal and mean pressure gradients in a rabbit model with calcific AS 56. What is more universally agreed upon however is that the uncoupling of nitric oxide synthase drives a major production of ROS 57.

Asymmetric dimethylarginine is a mediator of endothelial dysfunction and a competitive inhibitor of nitric oxide synthase 58. It induces oxidative stress via the upregulation of the renin-angiotensin system, with subsequent increases in ROS production 58. In fact, plasma asymmetric dimethylarginine levels are correlated with severity of AS 59.

Increased levels of DRP1 (dynamin-related protein 1), a mitochondrial protein that regulates mitochondrial fission, have been found in calcified human cardiovascular cells 60. DRP1 promotes osteogenic differentiation via oxidative stress, and its inhibition ameliorated calcification of VICs 60.

## Other Mechanisms of Oxidative Stress

Oxidative stress may activate matrix metalloproteinases in the aortic valve, which in turn may contribute to the degradation of the extracellular matrix and predispose the valve to calcification 55.

Uric acid is produced by the metabolism of purines via xanthine oxidase, another enzyme involved in oxidative processes, and can exert both antioxidant or oxidative effects depending on the cellular environment 61. In the context of degenerative AS, it contributes to endothelial dysfunction and accelerates the formation of oxLDLs; direct deposition of urate crystals in the aortic valve may accelerate the progression of degenerative AS too 6. While levels of serum uric acid are determined by several factors such as diet, catabolism and renal function, there is a positive correlation between serum uric acid and the severity of AS indices 6.

Similarly, the metabolism of serotonin by monoamine oxidase generates ROS such as superoxide 62. Serotonin, along with ROS, induces proliferation of VICs, leading to thickening and subsequent fibrosis and dysfunction of the valve; phenteramine, a monoamine oxidase inhibitor, in combination with fenfluramine, a serotonin reuptake inhibitor, can attenuate this effect 63.

Semicarbazide-sensitive amine oxidase (SSAO) is a mediator of oxidative stress and contributes to the development of atherosclerosis 64. The SSAO enzyme is involved in the production of hydrogen peroxide, and is upregulated in calcified aortic valves compared to normal valves 64. While serum SSAO levels are elevated in patients with traditional risk factors for atherosclerosis and degenerative AS - smoking, diabetes and obesity - SSAO levels within calcific valve tissue in degenerative AS are increased independent of these risk factors, and a positive correlation with the severity of AS is seen 64. SSAO is significantly associated with PARP-1 (poly[ADP-ribose] polymerase 1) in calcified valve tissue; PARP-1 correlates with AS severity and may directly contribute to soft tissue calcification in response to inflammation and oxidative stress 65. Immunohistochemical analysis of valve tissue found that SSAO is localised in proximity to calcified regions; with inhibition of SSAO, there was decreased calcification of valvular interstitial cells *in vitro* 64.

Soluble ST2 (interleukin 1 receptor-like 1) levels are positively correlated with the degree of oxidative stress and AS severity 66. It interferes with mitofusin-1 activity, disrupting mitochondrial fusion and oxidative phosphorylation capacity in VICs 67. This in turn may facilitate myocardial protein oxidation and inflammation. Furthermore, soluble ST2 can activate the osteogenic NF-kB pathway, and *in vitro* antioxidant treatment abolished these soluble ST2-mediated effects on oxidative stress and inflammation in cardiac fibroblasts 67.

## Oxidative Stress in Bicuspid Aortic Valves

Bicuspid aortic valves are categorised into different subtypes based on their phenotypes 68. Importantly, the mechanisms of progressive AS differ based on their subtypes. In Type I - fusion of the right and left coronary cusps - oxidative stress predominates, while in Type II - fusion of the right and non-coronary cusps - endothelial dysfunction is the main contributor 69. These however converge on the common pathway of inflammation, leading to subsequent valvular tissue remodelling and fibro-calcification.

As bicuspid aortic valves are less efficient at dissipating oscillatory shear stress compared to normal tricuspid aortic valves, accelerated endothelial dysfunction occurs. This stress induces ROS generation and lipid peroxidation 70, leading to an oxidative environment and further damaging the integrity of the extracellular matrix. Moreover, patients with bicuspid aortic valves have been demonstrated to have an abnormal antioxidant capacity, with an even greater lack of increased superoxide dismutase activity compared to patients with normal tricuspid valves 71.

Glutathione peroxidase-3 and sulfiredoxin-1 have also been found to be downregulated in endothelial cells in bicuspid aortic valves, compared to those in tricuspid aortic valves 72. Glutathione peroxidase-3 and sulfiredoxin are involved in the reduction of hydrogen peroxide, and the absence of their protective effects contributes to increased oxidative stress, leading to DNA damage and increased apoptosis 72.

## Lipid Lowering Therapies - A Key Target?

Given the key role of lipid peroxidation and infiltration, one of the most promising targets to combat degenerative AS was HMG-CoA reductase inhibitor therapy. One of the earlier animal studies involving rabbits with chronic hypercholesterolaemia demonstrated that atorvastatin inhibited bone mineralisation in the aortic valve by increasing the expression and activity of endothelial nitric oxide synthase 30. A small open-label trial that followed in 2007 showed that treatment with rosuvastatin slowed the haemodynamic progression of AS 73. However, large randomised controlled trials such as the SALTIRE 74, SEAS 75 and ASTRONOMER 76 failed to slow the progress of AS with statin therapy. In fact, in the ASTRONOMER trial, lipoprotein(a) and oxPL levels were increased with rosuvastatin compared to placebo, which may have abrogated the positive effects of reduced LDL cholesterol levels. Consequently, the American Heart Association/American College of Cardiology guidelines recommend against the use of statin therapy to prevent the haemodynamic progression of AS 3.

There is some promising data regarding Proprotein Convertase Subtilisin/Kexin Type 9 (PCSK9) inhibitors in the setting of AS. PCSK9 is an enzyme that binds to and targets the LDL receptor for degradation, and a genetic association study found that a loss-of-function mutation of the PCSK9 gene was linked to lower levels of LDL cholesterol, lipoprotein(a) and a reduced incidence of AS 77. These findings were echoed in a later genetic association study, which also found a higher level of PCSK9 expression in valvular tissues of patients with AS compared to those without 78. Furthermore, PCSK9 inhibition *in vitro* reduced calcium accumulation in VICs when they were exposed to a pro-osteogenic medium 78. In a secondary analysis of the FOURIER trial (an international, multicentre study which enrolled over 27,000 patients receiving statins for stable atherosclerotic disease and randomised them to receiving the PCSK9 inhibitor evolocumab versus a placebo), newly diagnosed or worsening AS, as well as aortic valve replacements, occurred in 63 patients. These events were associated with a higher level of lipoprotein(a), and there was a reduction in these events after the first year of treatment with evolocumab 79. This data appears to indicate that treatment with PCSK9 inhibitors may reduce the risk of AS development or progression, however this was a post-hoc analysis involving a small number of events, and further validation requires dedicated large randomised controlled trials.

Cholesterol ester transfer protein (CETP) is a plasma protein that regulates the transfer of cholesteryl esters from HDL cholesterol to other lipoproteins; its actions lead to reduction in the plasma levels of HDL cholesterol and thus reduces its cardioprotective effects 80. CETP is implicated in endothelial dysfunction, and induces ROS production in endothelial cells 80. CETP activity is also found to be increased in states of high oxidative stress, such as in type 2 diabetes mellitus, and may partially explain the predisposition of diabetics to cardiovascular disease 81,82.

While CETP inhibition has been found to also significantly lower lipoprotein(a) levels, three large clinical trials investigating the use of CETP inhibitors in cardiovascular disease were terminated early due to toxicity or futility 83-89. The only CETP inhibitor which showed a benefit was anacetrapib, which significantly reduced the incidence of major adverse coronary events compared to the placebo 90. However, the use of CETP inhibitors in the context of slowing or reversing AS has not been studied.

Synthetic oligonucleotides are biologics used to inactivate genes involved in disease processes. There are two main approaches: using an antisense RNA specific to the target disease-causing gene to disrupt its transcription, and using small interfering RNA fragments (siRNA) to cleave specific sequences in the mRNA transcript of the target disease-causing gene 91. Both result in the silencing of the disease-causing gene.

Antisense oligonucleotides targeting both apolipoprotein A and apolipoprotein B have shown promise in lowering LDL and plasma lipoprotein levels. In clinical trials, investigators found that antisense oligonucleotides against apolipoprotein A significantly reduced levels of lipoprotein(a) in a dose-dependent fashion 92,93. Antisense oligonucleotide therapy against apolipoprotein B resulted in significantly reduced lipoprotein(a) levels in multiple clinical trials involving patients with hypercholesterolaemia as well 94-100. Their use to slow or reverse AS has not been studied. Importantly, use of antisense oligonucleotide therapy against apolipoprotein B is associated with hepatic steatosis, which will likely limit their clinical usage 101.

## MicroRNAs - Targeting the Regulators of the Osteogenic Pathways

MicroRNAs (MiRNAs) are non-coding RNA sequences that are 21-23 nucleotides long, and regulate gene expression mainly via modulating mRNA translation during protein synthesis 52. They are involved in many body pathways such as the BMP2 pathway mentioned earlier, which is implicated in osteogenesis. MiRNAs bind to the 3' untranslated region of their target messenger RNA (mRNA) and mark them for destruction or by blocking its translation 52. Hence, manipulation of miRNAs may be a potential avenue for therapeutics in the treatment and prevention of degenerative AS.

Like in many other disease processes, miRNAs are implicated in degenerative AS as well. Levels of miR-26a, mIR-30b and mIR-195 were found to be reduced in stenotic bicuspid aortic valve tissues compared to insufficient ones 102. MiR-26a inhibits several calcification-related genes, while miR-30b reduces BMP2-induced osteogenic differentiation in VICs; the latter's upregulation was found to reduce the risk of aortic valve calcification 103. MiR-195 increases the expression of BMP2, RUNX2 and SMAD1, but also upregulates the expression of anti-calcification genes such as JAG2 (Jagged-2) and SMAD7 52.

MiR-141, another inhibitor of BMP2, is also found to be under-expressed in bicuspid valves when compared to tricuspid valves, and levels are even further reduced in stenotic bicuspid valves 104.

Another major miRNA involved in the calcification of the aortic valve is mIR-204, and it protects against calcification via the direct targeting of RUNX2 and SMAD4 105,106. Its expression was diminished in calcific aortic valve tissues compared to controls via the actions of TGF-β1 and BMP2, while its overexpression inhibited the osteogenic differentiation of VICs 106-108. In contrast, mIR-486 is upregulated in calcific aortic valves and targets a SMAD inhibitor known as SMURF2, thereby disinhibiting the SMAD pathway and resulting in the downregulation of mIR-204 109.

The effects of shear stress, which is the initiator of degenerative AS, are also modulated by miRNAs. Expression of miR-148-3p is increased in this abnormal haemodynamic environment, and upregulates the pro-osteogenic NF-kB pathway 110. Shear stress also increases the levels of mIR-214, which targets TWIST1 (twist-related protein 1), another inhibitor of osteogenic differentiation 111.

There are a number of circulating miRNAs whose levels are increased in the setting of degenerative AS. These may serve as biomarkers, however they appear to correlate more with the myocardial remodelling and dysfunction that are caused by degenerative AS 112. Moreover, variance in the levels of valvular miRNA does not correlate directly with the changes of their plasma levels 112. This may limit their use as potential biomarkers for the severity of AS before the onset of cardiac dysfunction.

MiRNAs have also been studied in relation to mitochondrial homeostasis, which is affected by oxidative stress and contributes to valvular calcification. An important protein in this process, DRP1, has been explored above, and it has been found that repression of miR-15a and miR-29a increases the levels of DRP1 in valvular tissues, leading to mitochondrial fission, subsequent apoptosis and contributing to calcification 113. Incidentally, expression of mIR-15a and miR-29a is reduced in stenotic valvular tissues 113.

Overexpression of protective miRNAs and inhibition of pro-osteogenic miRNAs may be potential strategies in the treatment of degenerative AS. MiRNA mimics and expression vectors can be used to increase the protective effects of select miRNAs, while sponge vectors and antisense oligonucleotides can be used to bind to and inhibit other miRNAs 114. A recent study in a murine model of calcific AS found that delivery of a miR-34a antagonist significantly attenuated the calcification of the aortic valves 115, which hopefully can be replicated in human trials in the future.

Major challenges in the development of possible therapies centring on miRNA manipulation lie in the *in vivo* stability of these therapeutics, as well as their delivery to target tissues. Furthermore, there is also imperfect base paring of miRNAs and target mRNAs, hence one type of mRNA can influence several pathways, which may lead to undesirable off-target effects 116. For example, miR-204 is involved in regulating mitochondrial transcription factor A in colon cancer 105.

## Other Potential Therapies

Overexpression of angiotensin converting enzyme (ACE) is seen in the valvular tissue of patients with AS, and its downstream product angiotensin II is a potent activator of the NF-kB pathway 7. Both ACE inhibitors and angiotensin receptor blockers (ARBs) have antioxidant effects, however usage of ACE inhibitors in the context of AS progression have had conflicting results 59,117-118. ARBs on the other hand, may be associated with less calcification of aortic valves and slower progression of AS 119. This difference may be explained by the presence of elevated levels of chymase in calcific aortic valve tissue, which, similar to ACE, converts angiotensin I to angiotensin II 120. Hence, the inhibition of ACE may be bypassed by the actions of chymase, resulting in the production of angiotensin II; while the effects of angiotensin II can be blocked by ARBs 7. The utility of ACE inhibitors and ARBs mainly lie in their antihypertensive effects that are independent of their antioxidant properties 118. They are used in the management of hypertension, which is commonly seen in patients with AS and is associated with a higher rate of ischaemic cardiovascular events and mortality 3. In addition, ACE inhibitors may reduce left ventricular remodelling in patients with AS 121.

A recent Korean study explored the anti-calcification effects of different DPP-4 inhibitors that were commercially available, and divided them into two groups - favourable (linagliptin and gemigliptin), and unfavourable (alogliptin, sitagliptin and vildagliptin) based on their heart and plasma concentrations after administration 122. Interestingly, they found that the use of linagliptin and gemigliptin in diabetic patients with mild-moderate AS were associated with a significantly lower rate of increase in the maximal transaortic velocity compared to the patients taking unfavourable DPP-4 inhibitors as well as those not taking DPP-4 inhibitors at all 122. This was a retrospective study, and whether these results can be replicated in a future prospective study remains to be seen. Table 2 summarises the literature for *in vitro* and animal models, while Table 3 summarises the clinical studies of therapies targeting the oxidative stress pathways in the treatment of AS 123-127.

## A Note on Aortic Sclerosis

Aortic sclerosis, a precursor stage to degenerative AS, is characterised by the thickening of the aortic valve. There is remodelling of the extracellular matrix, with or without biomineralization, and there is no alteration of the mechanical properties of the valve 128. Even in the absence of calcificafion, VICs in sclerotic aortic valves display an osteogenic phenotype, with upregulation of pro-osteogenic proteins such as bone morphogenetic protein 4 (BMP4) and Runx2 128. Further osteogenic differentiation and subsequent calcification are driven by both BMP4 and tensile stretch forces, leading to a vicious circle. It can thus be thought that aortic sclerosis and degenerative AS are on the same spectrum.

There is scant literature on the role of oxidative stress in the pathogenesis of aortic sclerosis. Patients with aortic sclerosis are largely asymptomatic and challenging to identify; additionally; sclerotic human aortic valves are generally not available to investigators as they are usually only surgically replaced when symptomatic or severe AS occurs 129.

One of the studies available explores glutathione, an endogenous antioxidant that helps counteract the effects of ROS. The homeostasis between the oxidised and reduced forms of glutathione is an indicator of oxidative stress, when the balanced is tipped in favour of the oxidised form of glutathione 130. ROS can react with the exposed cysteine residues of proteins in the aortic valve, which in turn can then be glutathionylated by reacting with the reduced form of glutathione 131. The affected proteins can be altered in structure and function and contribute to the development of aortic sclerosis. The same study found that patients with both atherosclerosis and aortic sclerosis had an imbalance of their systemic glutathione homeostasis, with elevated levels of glutathionylated proteins in their valves 131.

## Dietary Antioxidants

There is conflicting evidence regarding the benefits of dietary antioxidants such as vitamins in the prevention of other cardiovascular diseases such as ischaemic heart disease and atherosclerosis 132; suggesting that their use in the context of AS may result in similar outcomes.

There are several challenges and limitations to antioxidant therapy in general. Antioxidant therapy should ideally be initiated before disease onset, and should be used over a long term to allow its beneficial effects to emerge 133. Thus, their use in slowing the progression of AS may be limited, as the main role that oxidative stress plays appears to lie in the initiation of AS rather than its propagation.

The apparent discrepancy between the success of antioxidant therapy in animal and *in vitro* models versus their conflicting outcomes in human studies could be due to several factors. Firstly, *in vitro* experiments are isolated models testing a single variable, and do not reflect the complex biochemical environment in humans. There could be off-target effects of the antioxidant in question leading to an abrogation of its desired outcomes when tested in clinical trials, or even resulting in adverse effects. For example, a Cochrane review found that vitamin A and β-carotene consumption, two types of dietary antioxidants, was associated with an increase in all-cause mortality 134.

Secondly, lab feed that is provided to animals involved in experiments may be deficient in dietary antioxidants that are readily available in the normal human diet 135. Thus, the apparent positive effects of antioxidant administration may simply be the correction of an artificial vitamin deficiency. Finally, antioxidants themselves may become oxidised in the body and lose efficacy, and there could be difficulties in achieving therapeutic concentrations of the antioxidant while avoiding the threshold of toxicity 136.

Patients with AS typically also have other comorbidities such as hypertension and hypercholesterolaemia. It is possible that these patients are already benefiting from the antioxidant effects of their medications such as aspirin, statins, beta-blockers and ACE inhibitors/ARBs, and further antioxidant therapy may not have additional effects 136.

The innate intracellular activity of superoxide dismutase and other endogenous enzymes would outcompete most other antioxidant agents that are used intracellularly, hence intracellular scavenging of ROS as a therapeutic target is unlikely to have much benefit 135. Extracellularly however, superoxide dismutase mimics have higher kinetic rate constants compared to non-enzymatic reactions, and may be of potential use as a ROS scavenger in the context of AS 135. In fact, there is some early, exciting data of a recently approved superoxide dismutase mimic preventing aortic sclerosis in a murine model 137. Glutathione peroxidase mimics are also currently undergoing clinical trials in a range of diseases such as bipolar disorder and Meniere's disease 135. Perhaps these may be translated to human research for AS in the near future.

## Conclusion

Oxidative stress is a key denominator in the processes of inflammation, lipid infiltration and fibro-calcification in aortic stenosis, with distinct differentiation to the similar process of atherosclerosis. Statin therapy, while beneficial in the context of atherosclerosis, have demonstrated disappointing results in the slowing of AS progression. Other lipid lowering therapies such as PCSK9 inhibitors and CETP inhibitors may hold promise, however larger trials involving the use of these drugs are necessary. ARBs and DPP-4 inhibitors, which are commercially available prescription medications, also warrant further trials.

Manipulation of miRNAs involved in the pro-osteogenic pathways are also attractive targets, however this avenue is currently limited by the shortcomings in the stability and delivery of miRNA mimics, vectors and inhibitors, as well as their potential off-target effects.

Potential therapies that were studied for degenerative AS may have been commenced too late in the course of the disease to be effective. Hence, aortic sclerosis may provide an opportune target for pharmacological intervention. In fact, further osteogenic differentiation of VICs isolated from human aortic valves were amenable to reversal via antioxidant enzymes delivery 129.

As the pathways of oxidative stress in degenerative AS are complex, perhaps targeting a single component may not lead to clinically significant results. There is some evidence that targets outside the oxidative stress pathway, such as inhibition of cadherin 11, matrix metalloproteinases and even the use of direct oral anticoagulants (dabigatran, apixaban and rivaroxaban) may slow the progression of AS 138-139. Although oxidative stress in itself forms a major part of the pathophysiology of AS, it is not the full picture. Hence, a multimodal approach addressing several targets both inside and outside the oxidative stress pathway may be the way forward.

It is of worth to note that while antioxidants do provide a defence against oxidative stress, excessive antioxidants can lead to a phenomenon known as reductive stress, which, over time, is linked to pathological myocardial remodelling, development of diastolic dysfunction, and subsequent heart failure 140.

## Figures and Tables

**Figure 3:**
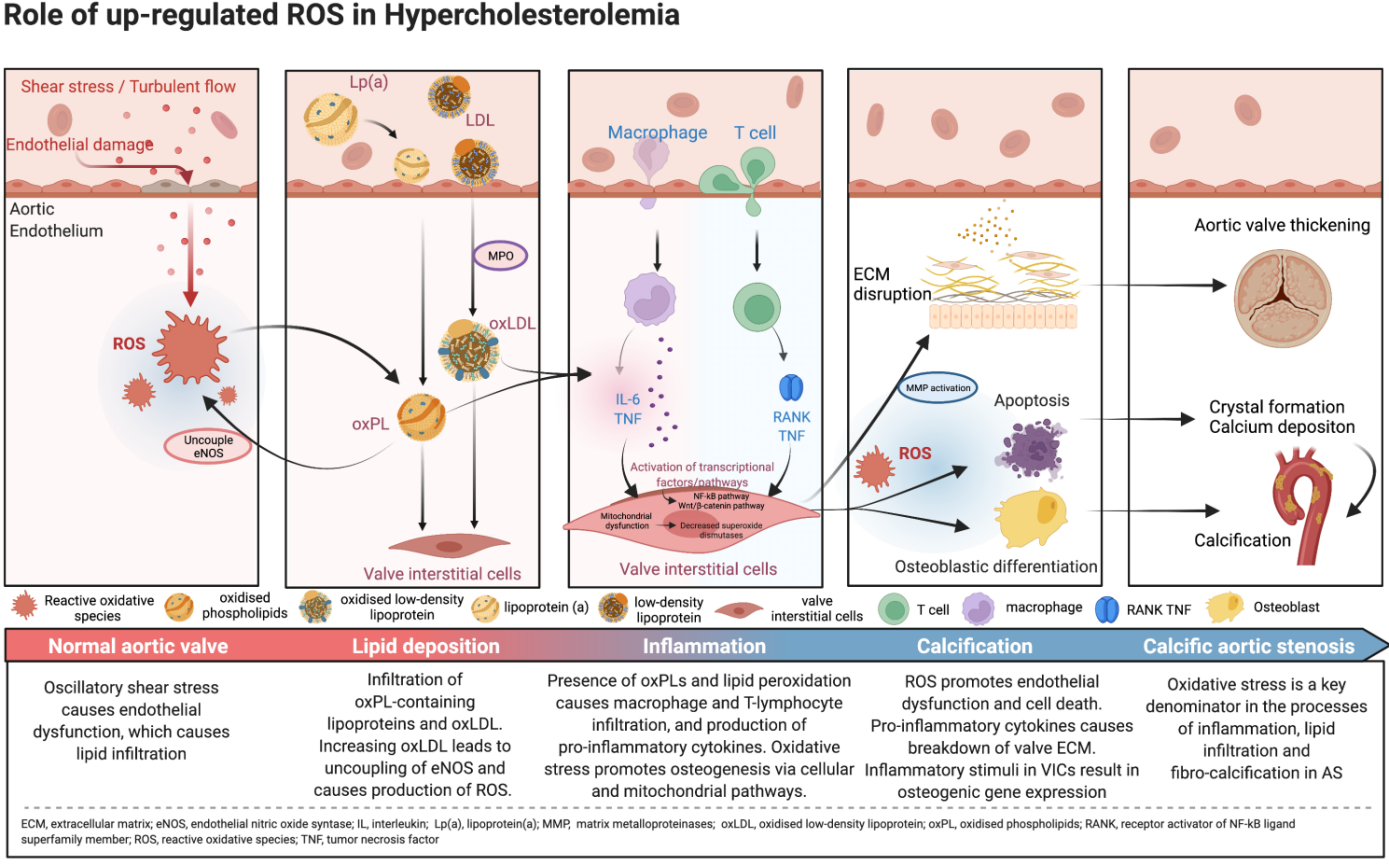
Central illustration. Pathophysiology and mechanistic pathways of oxidative stress in calcific aortic stenosis.

**Table 1:** Basic science and clinical studies demonstrating the association between oxidative stress and aortic stenosis.

First Author [Ref]	Year	Study Type	Key Findings
Arsenault BJ 34	2014	Cohort study (n=17,553)	Patients with Lp(a) levels in the top tertile had a higher risk of AS The genetic variant rs10455872, which is associated with higher levels of Lp(a), is also associated with increased risk of AS
Bosse K 50	2013	*In vitro* - porcine VICs	Nitric oxide prevents spontaneous calcification of porcine VICs Endothelial-derived NO signalling increases the expression of the NOTCH1 target gene
Bouchareb R 48	2015	*In vitro* - human VICs	Autotaxin is transported in the aortic valve by Lp(a) and promotes inflammation and mineralisation of the valve
Capoulade R 27	2015	Cohort study (n=220)	Elevated Lp(a) and OxPL levels are associated with faster AS progression and the need for aortic valve replacement
Choi B 46	2017	*In vitro* - human VICs	NO depletion in human VICs activates the NF-kB pathway, which promotes DPP-4 expression and subsequently induces osteogenic differentiation via reducing IGF-1 signalling
Côté C 35	2008	*Ex vivo* - human aortic valve tissue	Increased levels of circulating oxLDLs are associated with worse fibrocalcific remodelling of valvular tissue in AS
Demir B 6	2012	Cohort study (n=64)	There is a positive correlation between serum uric acid levels and AS severity Uric acid accelerates the formation of oxLDLs and may decrease NO levels
Hofmanis J 29	2019	Case control study (n=102)	AS severity is negatively correlated with levels of HDL cholesterol; higher MPO levels are negatively correlated with levels of HDL cholesterol as well MPO causes HDL cholesterol dysfunction via oxidation, reducing its protective effects
Kamstrup PR 36	2013	Cohort study (n=77,680)	Elevated Lp(a) levels and genotypes that increase plasma Lp(a) levels are associated with an increased risk of AS
Langsted A 77	2016	Cohort study (n=103,083)	PCSK9 loss-of-function mutation have lower levels of Lp(a) and reduced risk of AS
Li F 37	2015	*In vitro* - porcine VICs	oxLDLs induce VIC osteogenesis via activation of the receptor for advanced glycation end products (RAGE)
Liu H 57	2020	*In vitro* - human VICs	NADPH oxidase 2 is significantly increased in human calcific aortic valves
Matilla L 67	2019	*In vitro* - human VICs	Soluble ST2 disrupts mitochondrial fusion and oxidative phosphorylation capacity, as well as activates the osteogenic NF-kB pathway Soluble ST2 levels are positively correlated with oxidative stress and inflammation
Mercier N 64	2020	*In vitro* - human VICs	SSAO levels were positively correlated with increasing calcification SSAO inhibition decreased VIC calcification
Miller JD 16	2008	*In vitro* - superoxide and superoxide dismutase levels were measured in human aortic valves	Superoxide and hydrogen peroxide levels were increased in calcified regions of the aortic valve Superoxide dismutase activity and expression were reduced in calcified regions of the aortic valve
Mohty D 38	2008	*Ex vivo* - human aortic valve tissue	Valves with higher oxLDL content had higher levels of inflammatory cells, TNF-α and tissue remodelling
Nsaibia MJ 49	2016	Case-control study (n=300)	Lp(a) and oxPL levels were associated with higher autotaxin activity; patients with higher autotaxin and Lp(a) and oxPL levels had an increased risk of AS
Peña-Silva RA 62	2009	*Ex vivo* - human heart valves incubated with serotonin	Superoxide levels were increased after incubation with serotonin Inhibitors of flavin-oxidases or monoamine oxidase prevented the serotonin-induced increase in superoxide levels
Perrot N 78	2020	*Ex vivo* - human heart valves	PCSK9 expression was higher in valve tissue from patients with calcific AS compared to control patients PCSK9 levels were increased in human VICs incubated in an osteogenic medium, and a PCSK9 neutralising
		*In vitro* - human VICs incubated in an osteogenic medium	antibody significantly reduced calcium accumulation
Yu B 45	2017	*In vitro* - human VICs incubated in an osteogenic medium containing Lp(a) and OxPLs	Prolonged incubation of the VICs with Lp(a) significantly increased calcium deposition Calcium deposition was further augmented when VICs were incubated with both Lp(a) and OxPLs
Yu B 39	2018	*In vitro* - human VICs incubated in an osteogenic medium containing Lp(a) and OxPLs	Incubation of VICs with Lp(a) significantly increased ROS formation
Zeng Q 40	2014	*In vitro* - human VICs incubated incubated in an osteogenic medium	VICs incubated with oxLDLs had higher expression of the BMP-2 pathway and NOTCH1 signalling, with resultant increase in osteogenesis
Zheng KH 41	2019	Cohort study (n=145)	Patients with Lp(a) and OxPL levels in the top tertile had greater progression of valvular CT calcium score, faster haemodynamic progression on echocardiography, increased risk of aortic valve replacement and death

AS = aortic stenosis; DPP-4 = dipeptidyl peptidase-4; HDL = high density lipoproteins; IGF-1 = insulin-like growth factor 1; Lp(a) = lipoprotein(a); MPO = myeloperoxidase; NADPH = nicotinamide adenine dinucleotide phosphate; NF-kB = nuclear factor kappa light chain enhancer of activated B cells; NO = nitric oxide; oxLDL = oxidesed low density lipoproteins; oxPL = oxidised phospholipids; PCSK9 = proprotein convertase subtilisin/kexin type 9; ROS = reactive oxygen species; SSAO = semicarbazide-sensitive amine oxidase; ST2 = interleukin 1 receptor-like 1; TNF-α = tumour necrosis factor-alpha; VIC = valvular interstitial cells.

**Table 2:** Therapies targeting oxidative stress pathways in animal and *in vitro* models of aortic stenosis. BMP2 = bone morphogenetic protein 2; DPP-4 = dipeptidyl peptidase-4; DRP1 = dynamin-related protein 1; Lp(a) = lipoprotein(a); NADPH = nicotinamide adenine dinucleotide phosphate; NOS = nitric oxide synthase; oxLDL = oxidesed low density lipoproteins; oxPL = oxidised phospholipids; VIC = valvular interstitial cells.

First Author [Ref]	Year	Study Type	Key Findings
Bosse K 50	2013	*In vitro* - porcine VICs	Inhibition of NOS increases calcification
Choi B 46	2017	Mouse model - eNOS -/- Rabbit model with cholesterol-enriched diet	DPP-4 inhibition reduced aortic valve calcification Sitagliptin administration in a rabbit model with calcific aortic valve disease improved the rate of change in aortic valve area, transaortic peak velocity, and maximal and mean pressure gradients
Liberman M 54	2008	Rabbit model with cholesterol-enriched diet	*In vivo* treatment with lipolic acid, which aids the metabolism of hydrogen peroxide, decreases aortic valve calcification
Liu H 57	2020	*In vitro* - porcine VICs	Celastrol, which inhibits NADPH oxidase 2, significantly reduced calcification in porcine VICs
		Rabbit model with cholesterol-enriched diet	Celastrol, which inhibits NADPH oxidase 2, reduced aortic valve ROS production, fibrosis, calcification and AS severity
Nadlonek NA 123	2013	*In vitro* - human VICs	oxLDLs increased the expression of phosphate inorganic transporter 1 and BMP2, with resultant calcium and phosphate deposition. Inhibition of phosphate inorganic transporter 1 with phosphonoformate hexahydrate acid prevented oxLDL-induced BMP2 expression.
Rajamannan NM 30	2005	Rabbit model with chronic hypercholesterolaemia	Atorvastatin inhibits bone mineralisation in the aortic valve by increasing the protein expression and functional activity of endothelial NOS
Ratazzi M 44	2020	*In vitro* - bovine VICs stimulated to acquire a pro-calcific phenotype with endotoxin	L-Arginine, a precursor of NO, prevents osteogenic differentiation and reduces calcification
Rogers MA 58	2017	*In vitro* - human VICs	DRP1 inhibition attenuates VIC calcification
Zheng KH 41	2019	*In vitro* - human VICs incubated in an osteogenic medium with Lp(a) and OxPL	Incubation with the E06 monoclonal antibody against OxPL reduced osteogenic differentiation of VICs

**Table 3:** Clinical studies of therapies targeting the oxidative stress pathways in the treatment of aortic stenosis. ACE = acetylcholinesterase; ARB = angiotensin receptor blocker; AS = aortic stenosis; DPP-4 = dipeptidyl peptidase-4; Lp(a) = lipoprotein(a); PCSK9 = proprotein convertase subtilisin/kexin type 9.

First Author [Ref]	Year	Study Type	Key Findings
Bergmark BA 79	2020	Randomised controlled trial; post-hoc analysis (n = 27,564)	New/worsening AS and need for aortic valve replacement (AS events) occurred in 63 patients and were associated with elevated Lp(a) levels Overall hazard ratio for AS events patients on evolocumab (PCSK9 inhibitor) was significantly lower after a year of treatment
Capoulade R 119	2013	Observational study (n=338)	ARBs, but not ACE-inhibitors, were associated with slower AS progression in patients with concurrent hypertension and AS
Chan KL 76	2010	Randomised controlled trial (n=269)	Rosuvastatin 40mg once daily did not reduce the progression of AS in patients with known asymptomatic AS
Cowell SJ 74	2005	Randomised controlled trial (n = 155)	Atorvastatin 80mg once daily did not prevent the progression of AS nor induce regression in patients with known AS
Dichtl W 127	2008	Randomised controlled trial (n=47)	Atorvastatin 20mg once daily did not prevent the progression of AS in patients with known asymptomatic AS
Lee S 122	2020	Retrospective analysis (n=212)	In diabetic patients with mild-moderate AS, use of linagliptin or gemigliptin was associated with a slower rate of progression of maximal transaortic velocity as compared to patients on alogliptin, sitagliptin or vildagliptin, as well as patients not on any DPP-4 inhibitors
Moura LM 73	2007	Prospective open label (n=121)	Rosuvastatin 20mg once daily slowed the haemodynamic progression of AS in patients with known asymptomatic AS
O'Brien KD 126	2005	Retrospective analysis (n=123)	Treatment with ACE-inhibitors slowed the rate of aortic valve calcium accumulation
Rosenhek R 125	2004	Retrospective analysis (n=211)	Treatment with statins, but not ACE-inhibitors, slowed the rate of AS progression in patients with known AS
Rossebø AB 75	2008	Randomised controlled trial (n=1873)	Simvastatin and ezetimibe did not reduce the composite outcome of death from cardiovascular causes, aortic-valve replacement, nonfatal myocardial infarction, hospitalization for unstable angina pectoris, heart failure, coronary-artery bypass grafting, percutaneous coronary intervention, and nonhemorrhagic stroke in patients with asymptomatic AS
Shavelle DM 124	2002	Retrospective analysis (n=65)	Treatment with statins slowed progression of aortic valve calcium accumulation measured by electron-beam computed tomography

## References

[1] Yadgir S, Johnson CO, Aboyans V, Adebayo OM, Adedoyin RA, Afarideh M, et al; Global Burden of Disease Study 2017 Nonrheumatic Valve Disease Collaborators (2020). Global, Regional, and National Burden of Calcific Aortic Valve and Degenerative Mitral Valve Diseases, 1990-2017. Circulation.

[2] Akahori H, Tsujino T, Masuyama T, Ishihara M (2018). Mechanisms of aortic stenosis. J Cardiol.

[3] Writing Committee Members, Otto CM, Nishimura RA, Bonow RO, Carabello BA, Erwin JP 3rd (2021). 2020 ACC/AHA Guideline for the Management of Patients With Valvular Heart Disease: A Report of the American College of Cardiology/American Heart Association Joint Committee on Clinical Practice Guidelines. J Am Coll Cardiol.

[4] Chen JH, Simmons CA (2011). Cell-matrix interactions in the pathobiology of calcific aortic valve disease: critical roles for matricellular, matricrine, and matrix mechanics cues. Circ Res.

[5] Myasoedova VA, Ravani AL, Frigerio B, Valerio V, Moschetta D, Songia P (2018). Novel pharmacological targets for calcific aortic valve disease: Prevention and treatments. Pharmacol Res.

[6] Demir B, Caglar IM, Ugurlucan M, Ozde C, Tureli HO, Cifci S (2012). The relationship between severity of calcific aortic stenosis and serum uric acid levels. Angiology.

[7] Lindman BR, Clavel MA, Mathieu P, Iung B, Lancellotti P, Otto CM (2016). Calcific aortic stenosis. Nat Rev Dis Primers.

[8] Wypasek E, Natorska J, Mazur P, Kopytek M, Gawęda B, Kapusta P (2020). Effects of rivaroxaban and dabigatran on local expression of coagulation and inflammatory factors within human aortic stenotic valves. Vascul Pharmacol.

[9] Breyne J, Juthier F, Corseaux D, Marechaux S, Zawadzki C, Jeanpierre E (2010). Atherosclerotic-like process in aortic stenosis: activation of the tissue factor-thrombin pathway and potential role through osteopontin alteration. Atherosclerosis.

[10] Natorska J, Marek G, Hlawaty M, Sadowski J, Tracz W, Undas A (2011). Fibrin presence within aortic valves in patients with aortic stenosis: association with in vivo thrombin generation and fibrin clot properties. Thromb Haemost.

[11] Chew NW, Kong G, Ngiam JN, Phua K, Cheong C, Sia CH (2021). Comparison of Outcomes of Asymptomatic Moderate Aortic Stenosis With Preserved Left Ventricular Ejection Fraction in Patients ≥80 Years Versus 70-79 Years Versus <70 Years. Am J Cardiol.

[12] Larsson SC, Wolk A, Bäck M (2017). Alcohol consumption, cigarette smoking and incidence of aortic valve stenosis. J Intern Med.

[13] Larsson SC, Wolk A, Håkansson N, Bäck M (2017). Overall and abdominal obesity and incident aortic valve stenosis: two prospective cohort studies. Eur Heart J.

[14] Pawade TA, Newby DE, Dweck MR (2015). Calcification in Aortic Stenosis: The Skeleton Key. J Am Coll Cardiol.

[15] Pedriali G, Morciano G, Patergnani S, Cimaglia P, Morelli C, Mikus E (2020). Aortic Valve Stenosis and Mitochondrial Dysfunctions: Clinical and Molecular Perspectives. Int J Mol Sci.

[16] Miller JD, Chu Y, Brooks RM, Richenbacher WE, Peña-Silva R, Heistad DD (2008). Dysregulation of antioxidant mechanisms contributes to increased oxidative stress in calcific aortic valvular stenosis in humans. J Am Coll Cardiol.

[17] Plunde O, Bäck M (2021). Fatty acids and aortic valve stenosis. Kardiol Pol.

[18] Otto CM, Kuusisto J, Reichenbach DD, Gown AM, O'Brien KD (1994). Characterization of the early lesion of 'degenerative' valvular aortic stenosis. Histological and immunohistochemical studies. Circulation.

[19] Yin H, Xu L, Porter NA (2011). Free radical lipid peroxidation: mechanisms and analysis. Chem Rev.

[20] Heldmaier K, Stoppe C, Goetzenich A, Foldenauer AC, Zayat R, Breuer T (2018). Oxidation-Reduction Potential in Patients undergoing Transcatheter or Surgical Aortic Valve Replacement. Biomed Res Int.

[21] Marchandot B, Kibler M, Charles AL, Trinh A, Petit Eisenmann H, Zeyons F (2019). Does Transcatheter Aortic Valve Replacement Modulate the Kinetic of Superoxide Anion Generation?. Antioxid Redox Signal.

[22] Chew NWS, Phua K, Ho YJ, Zhang A, Lin N, Ngiam JN (2021). Prognostic Implications of Bicuspid and Tricuspid Aortic Valve Phenotype on Progression of Moderate Aortic Stenosis and Ascending Aorta Dilatation. Am J Cardiol.

[23] Sia CH, Ho JS, Chua JJ, Tan BY, Ngiam NJ, Chew N (2020). Comparison of Clinical and Echocardiographic Features of Asymptomatic Patients With Stenotic Bicuspid Versus Tricuspid Aortic Valves. Am J Cardiol.

[24] Zhong S, Li L, Shen X, Li Q, Xu W, Wang X (2019). An update on lipid oxidation and inflammation in cardiovascular diseases. Free Radic Biol Med.

[25] Youssef A, Clark JR, Koschinsky ML, Boffa MB (2021). Lipoprotein(a): Expanding our knowledge of aortic valve narrowing. Trends Cardiovasc Med.

[26] Cho KI, Sakuma I, Sohn IS, Jo SH, Koh KK (2018). Inflammatory and metabolic mechanisms underlying the calcific aortic valve disease. Atherosclerosis.

[27] Capoulade R, Chan KL, Yeang C, Mathieu P, Bossé Y, Dumesnil JG (2015). Oxidized Phospholipids, Lipoprotein(a), and Progression of Calcific Aortic Valve Stenosis. J Am Coll Cardiol.

[28] Schindhelm RK, van der Zwan LP, Teerlink T, Scheffer PG (2009). Myeloperoxidase: a useful biomarker for cardiovascular disease risk stratification?. Clin Chem.

[29] Hofmanis J, Hofmane D, Svirskis S, Mackevics V, Tretjakovs P, Lejnieks A (2019). HDL-C Role in Acquired Aortic Valve Stenosis Patients and Its Relationship with Oxidative Stress. Medicina (Kaunas).

[30] Rajamannan NM, Subramaniam M, Stock SR, Stone NJ, Springett M, Ignatiev KI (2005). Atorvastatin inhibits calcification and enhances nitric oxide synthase production in the hypercholesterolaemic aortic valve. Heart.

[31] Rajamannan NM (2011). Bicuspid aortic valve disease: the role of oxidative stress in Lrp5 bone formation. Cardiovasc Pathol.

[32] Vanhoutte PM, Zhao Y, Xu A, Leung SW (2016). Thirty Years of Saying NO: Sources, Fate, Actions, and Misfortunes of the Endothelium-Derived Vasodilator Mediator. Circ Res.

[33] Hong FF, Liang XY, Liu W, Lv S, He SJ, Kuang HB (2019). Roles of eNOS in atherosclerosis treatment. Inflamm Res.

[34] Arsenault BJ, Boekholdt SM, Dubé MP, Rhéaume E, Wareham NJ, Khaw KT (2014). Lipoprotein(a) levels, genotype, and incident aortic valve stenosis: a prospective Mendelian randomization study and replication in a case-control cohort. Circ Cardiovasc Genet.

[35] Côté C, Pibarot P, Després JP, Mohty D, Cartier A, Arsenault BJ (2008). Association between circulating oxidised low-density lipoprotein and fibrocalcific remodelling of the aortic valve in aortic stenosis. Heart.

[36] Kamstrup PR, Tybjærg-Hansen A, Nordestgaard BG (2014). Elevated lipoprotein(a) and risk of aortic valve stenosis in the general population. J Am Coll Cardiol.

[37] Li F, Zhao Z, Cai Z, Dong N, Liu Y (2015). Oxidized low-density lipoprotein promotes osteoblastic differentiation of valvular interstitial cells through RAGE/MAPK. Cardiology.

[38] Mohty D, Pibarot P, Després JP, Côté C, Arsenault B, Cartier A (2008). Association between plasma LDL particle size, valvular accumulation of oxidized LDL, and inflammation in patients with aortic stenosis. Arterioscler Thromb Vasc Biol.

[39] Yu B, Khan K, Hamid Q, Mardini A, Siddique A, Aguilar-Gonzalez LP (2018). Pathological significance of lipoprotein(a) in aortic valve stenosis. Atherosclerosis.

[40] Zeng Q, Song R, Ao L, Xu D, Venardos N, Fullerton DA (2014). Augmented osteogenic responses in human aortic valve cells exposed to oxLDL and TLR4 agonist: a mechanistic role of Notch1 and NF-κB interaction. PLoS One.

[41] Zheng KH, Tsimikas S, Pawade T, Kroon J, Jenkins WSA, Doris MK (2019). Lipoprotein(a) and Oxidized Phospholipids Promote Valve Calcification in Patients With Aortic Stenosis. J Am Coll Cardiol.

[42] Hutson HN, Marohl T, Anderson M, Eliceiri K, Campagnola P, Masters KS (2016). Calcific Aortic Valve Disease Is Associated with Layer-Specific Alterations in Collagen Architecture. PLoS One.

[43] Bäck M, Michel JB (2021). From organic and inorganic phosphates to valvular and vascular calcifications. Cardiovasc Res.

[44] Rattazzi M, Donato M, Bertacco E, Millioni R, Franchin C, Mortarino C (2020). l-Arginine prevents inflammatory and pro-calcific differentiation of interstitial aortic valve cells. Atherosclerosis.

[45] Yu B, Hafiane A, Thanassoulis G, Ott L, Filwood N, Cerruti M (2017). Lipoprotein(a) Induces Human Aortic Valve Interstitial Cell Calcification. JACC Basic Transl Sci.

[46] Choi B, Lee S, Kim SM, Lee EJ, Lee SR, Kim DH (2017). Dipeptidyl Peptidase-4 Induces Aortic Valve Calcification by Inhibiting Insulin-Like Growth Factor-1 Signaling in Valvular Interstitial Cells. Circulation.

[47] Nakanaga K, Hama K, Aoki J (2010). Autotaxin-an LPA producing enzyme with diverse functions. J Biochem.

[48] Bouchareb R, Mahmut A, Nsaibia MJ, Boulanger MC, Dahou A, Lépine JL (2015). Autotaxin Derived From Lipoprotein(a) and Valve Interstitial Cells Promotes Inflammation and Mineralization of the Aortic Valve. Circulation.

[49] Nsaibia MJ, Mahmut A, Boulanger MC, Arsenault BJ, Bouchareb R, Simard S (2016). Autotaxin interacts with lipoprotein(a) and oxidized phospholipids in predicting the risk of calcific aortic valve stenosis in patients with coronary artery disease. J Intern Med.

[50] Bosse K, Hans CP, Zhao N, Koenig SN, Huang N, Guggilam A (2013). Endothelial nitric oxide signaling regulates Notch1 in aortic valve disease. J Mol Cell Cardiol.

[51] Caira FC, Stock SR, Gleason TG, McGee EC, Huang J, Bonow RO (2006). Human degenerative valve disease is associated with up-regulation of low-density lipoprotein receptor-related protein 5 receptor-mediated bone formation. J Am Coll Cardiol.

[52] Nader J, Metzinger L, Maitrias P, Caus T, Metzinger-Le Meuth V (2020). Aortic valve calcification in the era of non-coding RNAs: The revolution to come in aortic stenosis management?. Noncoding RNA Res.

[53] Kim KM (1976). Calcification of matrix vesicles in human aortic valve and aortic media. Fed Proc.

[54] Liberman M, Bassi E, Martinatti MK, Lario FC, Wosniak J Jr, Pomerantzeff PM (2008). Oxidant generation predominates around calcifying foci and enhances progression of aortic valve calcification. Arterioscler Thromb Vasc Biol.

[55] Heistad DD, Wakisaka Y, Miller J, Chu Y, Pena-Silva R (2009). Novel aspects of oxidative stress in cardiovascular diseases. Circ J.

[56] Heistad DD (2008). Endothelial function in the time of the giants. J Cardiovasc Pharmacol.

[57] Liu H, Wang L, Pan Y, Wang X, Ding Y, Zhou C (2019). Celastrol Alleviates Aortic Valve Calcification Via Inhibition of NADPH Oxidase 2 in Valvular Interstitial Cells. JACC Basic Transl Sci.

[58] Rogers MA, Maldonado N, Hutcheson JD, Goettsch C, Goto S, Yamada I (2017). Dynamin-Related Protein 1 Inhibition Attenuates Cardiovascular Calcification in the Presence of Oxidative Stress. Circ Res.

[59] Ngo DT, Sverdlov AL, Horowitz JD (2012). Prevention of aortic valve stenosis: a realistic therapeutic target?. Pharmacol Ther.

[60] Cagirci G, Cay S, Canga A, Karakurt O, Yazihan N, Kilic H (2011). Association between plasma asymmetrical dimethylarginine activity and severity of aortic valve stenosis. J Cardiovasc Med (Hagerstown).

[61] Strazzullo P, Puig JG (2007). Uric acid and oxidative stress: relative impact on cardiovascular risk?. Nutr Metab Cardiovasc Dis.

[62] Peña-Silva RA, Miller JD, Chu Y, Heistad DD (2009). Serotonin produces monoamine oxidase-dependent oxidative stress in human heart valves. Am J Physiol Heart Circ Physiol.

[63] Mekontso-Dessap A, Brouri F, Pascal O, Lechat P, Hanoun N, Lanfumey L (2006). Deficiency of the 5-hydroxytryptamine transporter gene leads to cardiac fibrosis and valvulopathy in mice. Circulation.

[64] Mercier N, Pawelzik SC, Pirault J, Carracedo M, Persson O, Wollensack B (2020). Semicarbazide-Sensitive Amine Oxidase Increases in Calcific Aortic Valve Stenosis and Contributes to Valvular Interstitial Cell Calcification. Oxid Med Cell Longev.

[65] Nagy E, Caidahl K, Franco-Cereceda A, Bäck M (2012). Increased transcript level of poly(ADP-ribose) polymerase (PARP-1) in human tricuspid compared with bicuspid aortic valves correlates with the stenosis severity. Biochem Biophys Res Commun.

[66] Stundl A, Lünstedt NS, Courtz F, Freitag-Wolf S, Frey N, Holdenrieder S (2017). Soluble ST2 for Risk Stratification and the Prediction of Mortality in Patients Undergoing Transcatheter Aortic Valve Implantation. Am J Cardiol.

[67] Matilla L, Ibarrola J, Arrieta V, Garcia-Peña A, Martinez-Martinez E, Sádaba R (2019). Soluble ST2 promotes oxidative stress and inflammation in cardiac fibroblasts: an in vitro and in vivo study in aortic stenosis. Clin Sci (Lond).

[68] Fernandes SM, Sanders SP, Khairy P, Jenkins KJ, Gauvreau K, Lang P (2004). Morphology of bicuspid aortic valve in children and adolescents. J Am Coll Cardiol.

[69] Manno G, Bentivegna R, Morreale P, Nobile D, Santangelo A, Novo S (2019). Chronic inflammation: A key role in degeneration of bicuspid aortic valve. J Mol Cell Cardiol.

[70] Jones JA (2017). Oxidative stress in bicuspid aortic valve-related aortopathy: Hand-me-downs and yoga pants. J Thorac Cardiovasc Surg.

[71] Billaud M, Phillippi JA, Kotlarczyk MP, Hill JC, Ellis BW, St Croix CM (2017). Elevated oxidative stress in the aortic media of patients with bicuspid aortic valve. J Thorac Cardiovasc Surg.

[72] Poggio P, Songia P, Moschetta D, Valerio V, Myasoedova V, Perrucci GL (2019). MiRNA profiling revealed enhanced susceptibility to oxidative stress of endothelial cells from bicuspid aortic valve. J Mol Cell Cardiol.

[73] Moura LM, Ramos SF, Zamorano JL, Barros IM, Azevedo LF, Rocha-Gonçalves F (2007). Rosuvastatin affecting aortic valve endothelium to slow the progression of aortic stenosis. J Am Coll Cardiol.

[74] Cowell SJ, Newby DE, Prescott RJ, Bloomfield P, Reid J, Northridge DB, et al; Scottish Aortic Stenosis, Lipid Lowering Trial, Impact on Regression (SALTIRE) Investigators (2005). A randomized trial of intensive lipid-lowering therapy in calcific aortic stenosis. N Engl J Med.

[75] Rossebø AB, Pedersen TR, Boman K, Brudi P, Chambers JB, Egstrup K, et al; SEAS Investigators (2008). Intensive lipid lowering with simvastatin and ezetimibe in aortic stenosis. N Engl J Med.

[76] Chan KL, Teo K, Dumesnil JG, Ni A, Tam J; ASTRONOMER Investigators (2010). Effect of Lipid lowering with rosuvastatin on progression of aortic stenosis: results of the aortic stenosis progression observation: measuring effects of rosuvastatin (ASTRONOMER) trial. Circulation.

[77] Langsted A, Nordestgaard BG, Benn M, Tybjærg-Hansen A, Kamstrup PR (2016). PCSK9 R46L Loss-of-Function Mutation Reduces Lipoprotein(a), LDL Cholesterol, and Risk of Aortic Valve Stenosis. J Clin Endocrinol Metab.

[78] Perrot N, Valerio V, Moschetta D, Boekholdt SM, Dina C, Chen HY (2020). Genetic and In vitro Inhibition of PCSK9 and Calcific Aortic Valve Stenosis. JACC Basic Transl Sci.

[79] Bergmark BA, O'Donoghue ML, Murphy SA, Kuder JF, Ezhov MV, Ceška R (2020). An Exploratory Analysis of Proprotein Convertase Subtilisin/Kexin Type 9 Inhibition and Aortic Stenosis in the FOURIER Trial. JAMA Cardiol.

[80] Wanschel ACBA, Guizoni DM, Lorza-Gil E, Salerno AG, Paiva AA, Dorighello GG (2021). The Presence of Cholesteryl Ester Transfer Protein (CETP) in Endothelial Cells Generates Vascular Oxidative Stress and Endothelial Dysfunction. Biomolecules.

[81] Siewert S, Gonzalez II, Lucero RO, Ojeda MS (2015). Association of cholesteryl ester transfer protein genotypes with paraoxonase-1 activity, lipid profile and oxidative stress in type 2 diabetes mellitus: A study in San Luis, Argentina. J Diabetes Investig.

[82] Srivastava RAK (2018). Dysfunctional HDL in diabetes mellitus and its role in the pathogenesis of cardiovascular disease. Mol Cell Biochem.

[83] Stein EA, Raal F (2016). Future Directions to Establish Lipoprotein(a) as a Treatment for Atherosclerotic Cardiovascular Disease. Cardiovasc Drugs Ther.

[84] Thomas T, Zhou H, Karmally W, Ramakrishnan R, Holleran S, Liu Y (2017). CETP (Cholesteryl Ester Transfer Protein) Inhibition With Anacetrapib Decreases Production of Lipoprotein(a) in Mildly Hypercholesterolemic Subjects. Arterioscler Thromb Vasc Biol.

[85] Gencer B, Mach F (2020). Potential of Lipoprotein(a)-Lowering Strategies in Treating Coronary Artery Disease. Drugs.

[86] Handhle A, Viljoen A, Wierzbicki AS (2021). Elevated Lipoprotein(a): Background, Current Insights and Future Potential Therapies. Vasc Health Risk Manag.

[87] Barter PJ, Caulfield M, Eriksson M, Grundy SM, Kastelein JJ, Komajda M, et al; ILLUMINATE Investigators (2007). Effects of torcetrapib in patients at high risk for coronary events. N Engl J Med.

[88] Schwartz GG, Olsson AG, Abt M, Ballantyne CM, Barter PJ, Brumm J, et al; dal-OUTCOMES Investigators (2012). Effects of dalcetrapib in patients with a recent acute coronary syndrome. N Engl J Med.

[89] Lincoff AM, Nicholls SJ, Riesmeyer JS, Barter PJ, Brewer HB, Fox KAA, et al; ACCELERATE Investigators (2017). Evacetrapib and Cardiovascular Outcomes in High-Risk Vascular Disease. N Engl J Med.

[90] HPS3/TIMI55-REVEAL Collaborative Group, Bowman L, Hopewell JC, Chen F, Wallendszus K, Stevens W (2017). Effects of Anacetrapib in Patients with Atherosclerotic Vascular Disease. N Engl J Med.

[91] Langsted A, Nordestgaard BG (2019). Antisense Oligonucleotides Targeting Lipoprotein(a). Curr Atheroscler Rep.

[92] Tsimikas S, Viney NJ, Hughes SG, Singleton W, Graham MJ, Baker BF (2015). Antisense therapy targeting apolipoprotein(a): a randomised, double-blind, placebo-controlled phase 1 study. Lancet.

[93] Viney NJ, van Capelleveen JC, Geary RS, Xia S, Tami JA, Yu RZ (2016). Antisense oligonucleotides targeting apolipoprotein(a) in people with raised lipoprotein(a): two randomised, double-blind, placebo-controlled, dose-ranging trials. Lancet.

[94] Raal FJ, Santos RD, Blom DJ, Marais AD, Charng MJ, Cromwell WC (2010). Mipomersen, an apolipoprotein B synthesis inhibitor, for lowering of LDL cholesterol concentrations in patients with homozygous familial hypercholesterolaemia: a randomised, double-blind, placebo-controlled trial. Lancet.

[95] Akdim F, Visser ME, Tribble DL, Baker BF, Stroes ES, Yu R (2010). Effect of mipomersen, an apolipoprotein B synthesis inhibitor, on low-density lipoprotein cholesterol in patients with familial hypercholesterolemia. Am J Cardiol.

[96] Visser ME, Akdim F, Tribble DL, Nederveen AJ, Kwoh TJ, Kastelein JJ (2010). Effect of apolipoprotein-B synthesis inhibition on liver triglyceride content in patients with familial hypercholesterolemia. J Lipid Res.

[97] Akdim F, Tribble DL, Flaim JD, Yu R, Su J, Geary RS (2011). Efficacy of apolipoprotein B synthesis inhibition in subjects with mild-to-moderate hyperlipidaemia. Eur Heart J.

[98] Stein EA, Dufour R, Gagne C, Gaudet D, East C, Donovan JM (2012). Apolipoprotein B synthesis inhibition with mipomersen in heterozygous familial hypercholesterolemia: results of a randomized, double-blind, placebo-controlled trial to assess efficacy and safety as add-on therapy in patients with coronary artery disease. Circulation.

[99] McGowan MP, Tardif JC, Ceska R, Burgess LJ, Soran H, Gouni-Berthold I (2012). Randomized, placebo-controlled trial of mipomersen in patients with severe hypercholesterolemia receiving maximally tolerated lipid-lowering therapy. PLoS One.

[100] Thomas GS, Cromwell WC, Ali S, Chin W, Flaim JD, Davidson M (2013). Mipomersen, an apolipoprotein B synthesis inhibitor, reduces atherogenic lipoproteins in patients with severe hypercholesterolemia at high cardiovascular risk: a randomized, double-blind, placebo-controlled trial. J Am Coll Cardiol.

[101] Panta R, Dahal K, Kunwar S (2015). Efficacy and safety of mipomersen in treatment of dyslipidemia: a meta-analysis of randomized controlled trials. J Clin Lipidol.

[102] Nigam V, Sievers HH, Jensen BC, Sier HA, Simpson PC, Srivastava D (2010). Altered microRNAs in bicuspid aortic valve: A comparison between stenotic and insufficient valves. J. Heart Valve Dis.

[103] Zhang M, Liu X, Zhang X, Song Z, Han L, He Y (2014). MicroRNA-30b is a multifunctional regulator of aortic valve interstitial cells. J. Thorac. Cardiovasc. Surg.

[104] Yanagawa B, Lovren F, Pan Y, Garg V, Quan A, Tang G (2012). miRNA-141 is a novel regulator of BMP-2-mediated calcification in aortic stenosis. J Thorac Cardiovasc Surg.

[105] Ni WJ, Wu YZ, Ma DH, Leng XM (2018). Noncoding RNAs in Calcific Aortic Valve Disease: A Review of Recent Studies. J Cardiovasc Pharmacol.

[106] Gupta SK, Kumari S, Singh S, Barthwal MK, Singh SK, Thum T (2020). Non-coding RNAs: Regulators of valvular calcification. J Mol Cell Cardiol.

[107] Wang Y, Chen S, Deng C, Li F, Wang Y, Hu X (2015). MicroRNA-204 Targets Runx2 to Attenuate BMP-2-induced Osteoblast Differentiation of Human Aortic Valve Interstitial Cells. J Cardiovasc Pharmacol.

[108] Song R, Fullerton DA, Ao L, Zhao KS, Reece TB, Cleveland JC Jr (2017). Altered MicroRNA Expression Is Responsible for the Pro-Osteogenic Phenotype of Interstitial Cells in Calcified Human Aortic Valves. J Am Heart Assoc.

[109] Song R, Fullerton DA, Ao L, Zhao KS, Meng X (2017). An epigenetic regulatory loop controls pro-osteogenic activation by TGF-β1 or bone morphogenetic protein 2 in human aortic valve interstitial cells. J Biol Chem.

[110] Patel V, Carrion K, Hollands A, Hinton A, Gallegos T, Dyo J (2015). The stretch responsive microRNA miR-148a-3p is a novel repressor of IKBKB, NF-κB signaling, and inflammatory gene expression in human aortic valve cells. FASEB J.

[111] Li XF, Wang Y, Zheng DD, Xu HX, Wang T, Pan M, Shi JH, Zhu JH (2016). M1 macrophages promote aortic valve calcification mediated by microRNA-214/TWIST1 pathway in valvular interstitial cells. Am J Transl Res.

[112] Oury C, Servais L, Bouznad N, Hego A, Nchimi A, Lancellotti P (2016). MicroRNAs in Valvular Heart Diseases: Potential Role as Markers and Actors of Valvular and Cardiac Remodeling. Int J Mol Sci.

[113] Jan MI, Khan RA, Ali T, Bilal M, Bo L, Sajid A (2017). Interplay of mitochondria apoptosis regulatory factors and microRNAs in valvular heart disease. Arch Biochem Biophys.

[114] Nappi F, Iervolino A, Avtaar Singh SS, Chello M (2021). MicroRNAs in Valvular Heart Diseases: Biological Regulators, Prognostic Markers and Therapeutical Targets. Int J Mol Sci.

[115] Toshima T, Watanabe T, Narumi T, Otaki Y, Shishido T, Aono T (2020). Therapeutic inhibition of microRNA-34a ameliorates aortic valve calcification via modulation of Notch1-Runx2 signalling. Cardiovasc Res.

[116] van der Ven CF, Wu PJ, Tibbitt MW, van Mil A, Sluijter JP, Langer R (2017). In vitro 3D model and miRNA drug delivery to target calcific aortic valve disease. Clin Sci (Lond).

[117] de Nigris F, D'Armiento FP, Somma P, Casini A, Andreini I, Sarlo F (2001). Chronic treatment with sulfhydryl angiotensin-converting enzyme inhibitors reduce susceptibility of plasma LDL to in vitro oxidation, formation of oxidation-specific epitopes in the arterial wall, and atherogenesis in apolipoprotein E knockout mice. Int J Cardiol.

[118] Ogawa S, Kobori H, Ohashi N, Urushihara M, Nishiyama A, Mori T (2009). Angiotensin II Type 1 Receptor Blockers Reduce Urinary Angiotensinogen Excretion and the Levels of Urinary Markers of Oxidative Stress and Inflammation in Patients with Type 2 Diabetic Nephropathy. Biomark Insights.

[119] Capoulade R, Clavel MA, Mathieu P, Côté N, Dumesnil JG, Arsenault M (2013). Impact of hypertension and renin-angiotensin system inhibitors in aortic stenosis. Eur J Clin Invest.

[120] Helske S, Lindstedt KA, Laine M, Mäyränpää M, Werkkala K, Lommi J (2004). Induction of local angiotensin II-producing systems in stenotic aortic valves. J Am Coll Cardiol.

[121] Bull S, Loudon M, Francis JM, Joseph J, Gerry S, Karamitsos TD (2015). A prospective, double-blind, randomized controlled trial of the angiotensin-converting enzyme inhibitor Ramipril In Aortic Stenosis (RIAS trial). Eur Heart J Cardiovasc Imaging.

[122] Lee S, Lee SA, Choi B, Kim YJ, Oh SJ, Choi HM (2020). Dipeptidyl peptidase-4 inhibition to prevent progression of calcific aortic stenosis. Heart.

[123] Nadlonek NA, Lee JH, Weyant MJ, Meng X, Fullerton DA (2013). ox-LDL induces PiT-1 expression in human aortic valve interstitial cells. J Surg Res.

[124] Shavelle DM, Takasu J, Budoff MJ, Mao S, Zhao XQ, O'Brien KD (2002). HMG CoA reductase inhibitor (statin) and aortic valve calcium. Lancet.

[125] Rosenhek R, Rader F, Loho N, Gabriel H, Heger M, Klaar U (2004). Statins but not angiotensin-converting enzyme inhibitors delay progression of aortic stenosis. Circulation.

[126] O'Brien KD, Probstfield JL, Caulfield MT, Nasir K, Takasu J, Shavelle DM (2005). Angiotensin-converting enzyme inhibitors and change in aortic valve calcium. Arch Intern Med.

[127] Dichtl W, Alber HF, Feuchtner GM, Hintringer F, Reinthaler M, Bartel T (2008). Prognosis and risk factors in patients with asymptomatic aortic stenosis and their modulation by atorvastatin (20 mg). Am J Cardiol.

[128] Poggio P, Sainger R, Branchetti E, Grau JB, Lai EK, Gorman RC (2013). Noggin attenuates the osteogenic activation of human valve interstitial cells in aortic valve sclerosis. Cardiovasc Res.

[129] Branchetti E, Sainger R, Poggio P, Grau JB, Patterson-Fortin J, Bavaria JE (2013). Antioxidant enzymes reduce DNA damage and early activation of valvular interstitial cells in aortic valve sclerosis. ]. Arterioscler Thromb Vasc Biol.

[130] Asensi M, Sastre J, Pallardo FV, Lloret A, Lehner M, Garcia-de-la Asuncion J (1999). Ratio of reduced to oxidized glutathione as indicator of oxidative stress status and DNA damage. Methods Enzymol.

[131] Valerio V, Myasoedova VA, Moschetta D (2019). Impact of Oxidative Stress and Protein S-Glutathionylation in Aortic Valve Sclerosis Patients with Overt Atherosclerosis. J Clin Med.

[132] Whayne TF, Saha SP, Mukherjee D (2016). Antioxidants in the Practice of Medicine; What Should the Clinician Know?. Cardiovasc Hematol Disord Drug Targets.

[133] Mitra S, Deshmukh A, Sachdeva R, Lu J, Mehta JL (2011). Oxidized low-density lipoprotein and atherosclerosis implications in antioxidant therapy. Am J Med Sci.

[134] Bjelakovic G, Nikolova D, Gluud LL, Simonetti RG, Gluud C (2012). Antioxidant supplements for prevention of mortality in healthy participants and patients with various diseases. Cochrane Database Syst Rev.

[135] Forman HJ, Zhang H (2021). Targeting oxidative stress in disease: promise and limitations of antioxidant therapy. Nat Rev Drug Discov.

[136] Malekmohammad K, Sewell RDE, Rafieian-Kopaei M (2019). Antioxidants and Atherosclerosis: Mechanistic Aspects. Biomolecules.

[137] Anselmo W, Branchetti E, Grau JB, Li G, Ayoub S, Lai EK (2018). Porphyrin-Based SOD Mimic MnTnBu OE -2-PyP5+ Inhibits Mechanisms of Aortic Valve Remodeling in Human and Murine Models of Aortic Valve Sclerosis. J Am Heart Assoc.

[138] Natorska J, Kopytek M, Undas A (2021). Aortic valvular stenosis: Novel therapeutic strategies. Eur J Clin Invest.

[139] Lindman BR, Sukul D, Dweck MR, Madhavan MV, Arsenault BJ, Coylewright M (2021). Evaluating Medical Therapy for Calcific Aortic Stenosis: JACC State-of-the-Art Review. J Am Coll Cardiol.

[140] Shanmugam G, Wang D, Gounder SS, Fernandes J, Litovsky SH, Whitehead K (2020). Reductive Stress Causes Pathological Cardiac Remodeling and Diastolic Dysfunction. Antioxid Redox Signal.

